# Use of Bayesian methods to model the SF-6D health state preference based data

**DOI:** 10.1186/s12955-018-1068-7

**Published:** 2018-12-18

**Authors:** Samer A. Kharroubi

**Affiliations:** Department of Nutrition and Food Sciences, Faculty of Agricultural and Food Sciences, American University of Beirut, P.O.BOX: 11-0236, Riad El Solh 1107-2020, Beirut, Lebanon

**Keywords:** Preference based health states, SF-6D, Cost-utility analysis, Bayesian methods, MCMC

## Abstract

**Background:**

Conventionally, models used for health state valuation data have been frequentists. Recently a number of researchers have investigated the use of Bayesian methods in this area. The aim of this paper is to put on the map of modelling a new approach to estimating SF-6D health state utility values using Bayesian methods. This will help health care professionals in deriving better health state utilities of the original UK SF-6D for their specialized applications.

**Methods:**

The valuation study is composed of 249 SF-6D health states valued by a representative sample of the UK population using the standard gamble technique. Throughout this paper, we present four different models, including one simple linear regression model and three random effect models. The predictive ability of these models is assessed by comparing predicted and observed mean SF-6D scores, R^2^/adjusted R^2^ and RMSE. All analyses were carried out using Bayesian Markov chain Monte Carlo (MCMC) simulation methods freely available in the specialist software WinBUGS.

**Results:**

The random effects model with interaction model performs best under all criterions, with mean predicted error of 0.166, R^2^/adjusted R^2^ of 0.683 and RMSE of 0.218.

**Conclusions:**

The Bayesian models provide flexible approaches to estimate mean SF-6D utility estimates, including characterizing the full range of uncertainty inherent in these estimates. We hope that this work will provide applied researchers with a practical set of tools to appropriately model outcomes in cost-effectiveness analysis.

## Background

The use of the preference-based measures of health related quality of life (HRQoL) has been burgeoning over the past years in the field of health economics as means to calculate the quality adjusted life years (QALYs) to employ them in cost-effectiveness analyses (CEA) across the different available treatments, which gained their essentiality due to the booming power, expenses and variety of modern medicine. The utility, defined as the reference to a measure of HRQoL in health economics, may be commonly captured by adopting a standardized multi-attribute utility (MAU) questionnaire with pre-existing utility weights derived from the general population, and the overall term “health related utility” refers to the measuring of consequence to a person for being in a specific state of health.

One difficulty with constructing such a measure is the complex nature of a ‘state of health’. Several MAU questionnaires have been developed to measure HRQOL and one of the most widely used is the SF-36 [1]. Health is a multidimensional thing, and the SF-36 describes a patient’s state of health across eight dimensions by their answers to 36 multiple-choice questions. Each question represents a particular aspect of health, and the patient describes how good or bad their health is by using a discrete response scale in that dimension. Constructing a utility measure to describe the overall quality of life for such a complex multidimensional descriptive system that defines millions of potential health states is a very difficult task, and health economists have instead based their utility measures on simpler health state descriptions, including the EQ-5D [2, 3], Health Utilities Index (HUI) [4], HUI3 [5], 15D [6, 7], QWB [8], the Assessment of Quality of Life (AQoL) [9], and the SF-6D [10, 11].

However, a couple of pivotal hurdles are caused by these instruments, where the first is imposed by their defined large number of unique health states and the resulting need to adopt the valuation of a subset of possible sates in order to model health state values, and the second hurdle is induced by their complex nature rendering their statistical modelling quite challenging.

In spite of these obstacles, Brazier et al. [11] witnessed some success with the modelling of the SF-6D data, while they unearthed the issues related to non-monotonicity, implying a lower value predicted for better states than worse states, and to the size and the methodical pattern of prediction errors, where some values of bad health states are over predicted while values of good health states are under predicted. This paper presents an alternative Bayesian approach for modelling health state preference data for handling these problems and demonstrates how regression analysis can be implemented relatively quickly and easily using Bayesian methods. It also provides important evidence on the advantages of this approach to modelling health state preference data, especially in the out of sample validation. Further, the paper shows that the Bayesian framework is more flexible in characterizing inputs to regression models and more comprehensive in characterizing the uncertainty in the model outputs.

The second section of this paper provides a brief description of the SF-6D valuation study and the adapted data. The models used for the analysis including assessment of model complexity and fit are also outlined. The next section illustrates the application of the proposed method to the analysis of SF-6D data. The last section concludes with a discussion of the results and their implications for future use of the SF-6D and modelling in CEA.

## Methods

### The SF-6D

As a generic measure of health, the SF-6D has been derived from the original health based preference SF-36 [1], resulting in a total of six dimensions (each between four and six levels): *physical functioning, role limitations, social functioning, pain, mental health, and vitality*. To define an SF-6D health state, the respondent has to choose the level which best suits him/her from each dimension, starting with *physical functioning* and ending with *vitality*. In order to minimize loss of descriptive information, the SF-6D has been constructed from a subset of 11 items selected from the initial SF-36, allowing the determination of 18,000 health states [12]. Level 1 in each dimension indicates no loss of health while levels 2 to 6 refer to a certain loss of health. Hence, a health state of 111,111 represents perfect health, while health state 645,655 is the worst health state, or the “pits”.

### Study design

The basic design of the survey was that a sample of 249 health states defined by the SF-6D was valued by a representative sample of the UK general population (*n* = 836). Each respondent was asked to rank, and then value, six of these states using a variant of the standard gamble (SG) technique.

#### Selection of respondents

The purpose of sampling was to ensure the sample reflects the variability of the population in terms of characteristics, such as age, socio-economic status, and level of education. The sample was drawn using a two-stage cluster random selection design. The primary units were postcode sectors stratified by percentage of households. Fifty-one postcode sectors were selected, and addresses were randomly selected from each of these, resulting in 1445 potential interviews. More on this is available in Brazier et al. [11].

#### Selection of health states

The 249 health states were selected in two ways. One part of the sample was identified using an orthogonal design (by applying the Orthoplan procedure of SPSS) which generates 49 health states to estimate an additive model. A further 200 states were selected using a stratified random sampling method to ensure a balance of mild, moderate and severe states. More on this is available in Brazier et al. [11].

#### Interviews

Each respondent was asked to rank and value six health states using the McMaster ‘ping pong’ variant of the SG. The SG technique asked the respondents to value five of the six SF-6D health states against the perfect health state and the “pits”. Respondents were then asked in the sixth SG question to value ‘pits’. Depending on whether they thought this state was better or worse than death they would be asked to consider one of the following choices: (i) the certain prospect of being in the “pits” state and the uncertain prospect of full health or immediate death; or (ii) the certain prospect of death and the uncertain prospect of full health or the “pits” state [12]. The chances of the best outcome occurring is varied until the respondent is indifferent between the certain and uncertain prospects. The negative of the indifference probability of the best outcome, having the effect of bounding negative values at − 1, has been assigned to states valued worse than death [13]. Then, the other 5 health states were chained onto the zero to one scale, where 0 is given to states perceived equivalent to being dead, and 1 is given to perfect health [11]. These adjusted SG values form the dependent variable (*y*) in the models discussed below.

### Study sample

Out of the 1445 addresses contacted for the interview, 167 proved to be ineligible.^1^ Of these 1278 (1445–167) usable addresses 836 respondents agreed to participate in the face-to-face interviews (a 65% response rate).^2^ Respondents were found to be representative of the national population in terms of the distribution by age group, education and social class. Out of these 836 respondents in the face-to-face interviews, 130 were excluded from the analysis since they failed to value the pits state and it was therefore not possible to generate an adjusted SG value for them, and another 9 were excluded for not valuing two or more health states. A further 86 respondents who gave the same valuation for each of the five states were also excluded, leaving 611 (836–130–9-86) respondents data for analysis [10]. Each of the 611 respondents made 6 SG valuations giving 3666 valuations. Of these, 148 were missing from 117 respondents, so 3518 (3666–148) SG valuations across 249 health states were finally included in the analysis. Each of those health states has been valued in average 15 times, with mean health state values ranging from 0.10 (health state 535,645) to 0.99 (state 111,111) and standard deviations ranging from 0.02 (111111) to 0.61 (434654). Median health state values usually exceeded mean values, reflecting the negative skewness of the data. Negative observations (suggesting states worse than death) were comparatively rare (245/3518) and only 20/3518 health states were given a value of 1.0, while 23% of the observations lie between 0.9 and 1.0 [11].

The data set is available upon requested from Prof John Brazier (j.e.brazier@sheffield.ac.uk), who was investigator in the original SF-6D valuation survey, at the University of Sheffield, UK.

### Modelling

As mentioned earlier, the SF-6D could describe 18,000 possible health states, and the empirical survey could obtain valuations for a small subset. Hence, the aim of modelling is to estimate health state utility values for all states. We developed a series of Bayesian predictions using four different approaches, including one simple linear regression model and three random effect (RE) models. In each of the four models, the utility weight from the SF-6D is considered as our dependent variable. Our independent variables were dummy explanatory variables for each level above 1 from each of the six dimensions of the SF-6D, in addition to extra dummy variable to account for interactions between the levels of different dimensions. We shall discuss these in more detail when considering the models below.

#### Bayesian methods

Bayesian methods [14, 15] allow the incorporation of information external to the observed data into the analysis. Such information is specified in a prior distribution and is combined with the observed data to produce a posterior distribution on which inferences are based. The incorporation of informative a priori beliefs is not a requirement because “vague” or “noninformative” priors can be used that provide very little or no information relative to the data. As the focus of this paper is not on the incorporation of prior information, all prior distributions placed on model parameters are assumed to be “vague”.

#### MCMC methods

The computation of the posterior distributions for parameters in a Bayesian model are often complex. MCMC methods [16] are computer-intensive methods that allow one to simulate from the posterior distribution, without having to explicitly calculate the posterior distribution. One method, Gibbs sampling, can be used to estimate posterior distributions by drawing sample values randomly from the full conditional distributions of each parameter conditional on all others and the data. Historically, sampling from one conditional distribution required considerable amount of computer programming. Fortunately, the necessary computation routines are now freely available in the software package WinBUGS (https://www.mrc-bsu.cam.ac.uk/software/bugs/) [17] which only requires the actual model to be specified. WinBUGS is used for all the analyses in this paper and the relevant code is available from the author.

#### Model development

*Model 1.* The linear regression model is defined by the following:

1 $$ {Y}_i={\beta}_0+{\beta}_1{X}_{1i}+{\beta}_2{X}_{2i}+\dots +{\beta}_k{X}_{ki}+{\varepsilon}_i, $$

where *Y*_*i*_ is the utility weight from the SF-6D of patient *i,* the subscript *i* refers to the observation and so it runs from 1 to the total number of observations, which is 3518 for this data set; *X*_*ki*_ indicates values for the *k* covariates for individual *i*; the βs are the regression parameters and *ε*_*i*_ is a random error term associated with each observation.

Note that *X* is a vector of dummy explanatory variables (*X*_*δλ*_) for each level λ > 1 of dimension δ of the SF-6D. For example, *X*_32_ denotes dimension δ = 3 (social functioning), level λ = 2 (health limits social activities a little of the time). For any given health state, *X*_*δλ*_ is defined as:

*X*_*δλ*_ = 1 if, for this state, dimension δ is at level λ.

*X*_*δλ*_ = 0 if, for this state, dimension δ is not at level λ.

In all, there are 25 of these terms, with level λ = 1 on each dimension acts as the baseline for each dimension.

Model 1, which uses the OLS model in (1), assumes that the error term *ε*_*i*_ in Eq. 1 is normally distributed with constant variance (homoscedastic): *ε*_*i*_  ∼  *N*(0, *σ*^2^) but can be relaxed to allow for non-constant variance (heteroscedasticity). This assumption means that the 3518 observations from 611 respondents are treated as though 3518 respondents provided them. For this model, we assumed that all the observed SF-6D utility values are drawn from a normal distribution, with the conditional mean (*μ*_*i*_) a function of each of the 25 levels of the SF-6D, as seen in Table 1.

**Table 1:** Specifications of the four models (simple linear regression and RE)

Model	Conditional Distribution	Specification of the mean
M1- Linear regression	*Y*_*i*_~*N*(*μ*_*i*_, σ^2^) *ε*_*i*_~*N*(0, σ^2^)	*μ*_*i*_ = *β*_0_ + *Para*_*i*_
M2- Random effect	*Y*_*ij*_~*N*(*μ*_*ij*_, σ^2^) ${u}_i\sim N\left(0,{\upsigma}_u^2\right)$ ${e}_{ij}\sim N\left(0,{\upsigma}_e^2\right)$	*μ*_*ij*_ = *β*_0_ + *Para*_*ij*_ + *u*_*i*_
M3- Random effect: intercept forced to unity	*Y*_*ij*_~*N*(*μ*_*ij*_, σ^2^) ${u}_i\sim N\left(0,{\upsigma}_u^2\right)$ ${e}_{ij}\sim N\left(0,{\upsigma}_e^2\right)$	*μ*_*ij*_ = *Para*_*ij*_ + *u*_*i*_
M4- Random effect: intercept forced to unity and inclusion of most	*Y*_*ij*_~*N*(*μ*_*ij*_, σ^2^) ${u}_i\sim N\left(0,{\upsigma}_u^2\right)$ ${e}_{ij}\sim N\left(0,{\upsigma}_e^2\right)$	*μ*_*ij*_ = *Para*_*ij*_ + *β*_*most*_*most*_*ij*_ + *u*_*i*_

*Para*_*ij*_ = *β*_*PF*2_*PF*2_*ij*_ + *β*_*PF*3_*PF*3_*ij*_ + *β*_*PF*4_*PF*4_*ij*_ + *β*_*PF*5_*PF*5_*ij*_ + *β*_*PF*6_*PF*6_*ij*_ + *β*_*RL*2_*RL*2_*ij*_ + *β*_*RL*3_*RL*3_*ij*_ + *β*_*RL*4_*RL*4_*ij*_ + *β*_*SF*2_*SF*2_*ij*_ + *β*_*SF*3_*SF*3_*ij*_ + *β*_*SF*4_*SF*4_*ij*_ + *β*_*SF*5_*SF*5_*ij*_ + *β*_*PAIN*2_*PAIN*2_*ij*_ + *β*_*PAIN*3_*PAIN*3_*ij*_ + *β*_*PAIN*4_*PAIN*4_*ij*_ + *β*_*PAIN*5_*PAIN*5_*ij*_ + *β*_*PAIN*6_*PAIN*6_*ij*_ + *β*_*MH*2_*MH*2_*ij*_ + *β*_*MH*3_*MH*3_*ij*_ + *β*_*MH*4_*MH*4_*ij*_ + *β*_*MH*5_*MH*5_*ij*_ + *β*_*VIT*2_*VIT*2_*ij*_ + *β*_*VIT*3_*VIT*3_*ij*_ + *β*_*VIT*4_*VIT*4_*ij*_ + *β*_*VIT*5_*VIT*5_*ij*_

*Model 2.* The Random Effects (RE) model (Model 2) acknowledges that the error term may not be independent of the respondent, and therefore separates out within and between respondent error terms, namely:

2 $$ {u}_i+{e}_{ij} $$

where *u*_*i*_ is respondent specific variation, which is assumed to be random across individual respondents, *eij* is an error term for the *j*th health state valuation of the *i*th individual, and this is assumed to be random across observations. This model also assumes that the allocation of health states to respondents is random i.e. cov*(ui, eij)* = 0.

*Model 3.* There are strong theoretical arguments for restricting the intercept to unity. The adjusted SG value for each state has been estimated according to the axioms of expected utility theory by assuming the best health state defined by the SF-6D (i.e. state 111,111) is to equal one and death is equal to zero. For state 111,111 to hold any other value would change the scale. Furthermore, for use in cost utility analysis it is necessary to assume that health state 111,111 is equivalent to full health and hence has a value of one. The best way to ensure health state 111,111 has a value of one is to restrict the intercept to unity [11]. Thus, we construct the third model to align with this idea. Model 3 has the same equation as in model 2 with the simple exclusion of the intercept.

*Model 4.* A possible option, was to base our study on the simple orthogonal design of the survey, however, we would be effectively limiting ourselves to the main effects. Yet, a common application in health state valuation modelling is to study the interactions between the dimensions [11, 18], which amount to 6 dimensions for this data set. We went through the process of looking up interactions in the SF-6D data set, and we ended up with a hefty number of possible interactions. Additionally, if they were all to be modelled, then this would require a valuation data on a larger sample of health states than the one we have on hands, and the possibility of finding statistically significant interactions would be solely dependent on the play of chance. However, other studies have found some significant interactions in the type of modelling of our concern [11, 18]. Accordingly, we based the choice of interactions for our modelling on their findings. Specifically, extreme level dummies were created to represent the number of times a health state contains dimensions at the extreme ends of the scale. Most severe is defined as levels 4 to 6 for *Physical Functioning* (PF), levels 3 and 4 for *Role Limitations* (RL), levels 5 and 6 for *Pain* (PAIN), and levels 4 and 5 for the three dimensions *Social Functioning* (SF), *Mental Health* (MH) and *Vitality* (VIT).

Hence, model 4 is based on the same Equation as in model 3 with the inclusion of an additional variable, ‘most’, to account for interactions between the levels of different dimensions, which takes the value of 1 if any dimension in the health state is at one of the most severe levels, and 0 otherwise. See Table 1 for more detailed information about this and the rest of the models.

To this end, the prior distributions for all the regression coefficients are defined to be Normal (0, 10^6^), i.e., centered on 0 with a large variance so as to be relatively noninformative. When declaring normal distributions in WinBUGS, instead of defining variances, precisions (the reciprocal of the variance) are specified (to make the specification of priors more straightforward); hence, τ = 1/σ^2^. Because τ is the inverse of a variance parameter (which cannot go negative), a different prior is required; the common choice of Gamma (0.001, 0.001) is used, with the intention of being minimally informative. That is, prior distributions were specified as follows:

$$ {\beta}_0,\dots {\beta}_k\sim N\left(0,{10}^6\right),\kern0.5em {\sigma}^2\sim \mathrm{InverseGamma}\left(0.001,0.001\right) $$

See [19] for further discussion regarding choice of noninformative prior distributions.

#### Model estimation

After conducting some initial runs to test the data, and based on the Gelman and Rubin diagnostic for assessment [20], we decided on the need to have some initial runs of 1000 iterations as “burn in” in order to reach convergence, noting that these runs were excluded from the final results [16]. First, we formed two parallel chains from broadly spread initial values, and then we monitored the variance of the ratio of the within-chain to between-chain which reached convergence at about one. Following that, a further 10,000 iterations were run for the purposes of predictions and parameters estimation. Albeit, the figures obtained depend on the degree of the movement smoothness of the sampler around the sample space, hence, they are not constant throughout all applications.

#### Model reliability and validation

The performance of all models was compared by calculating the proportion of variance they explained in the sample, using the unadjusted *R*^2^ statistic, where [21, 22]

3 $$ {R}^2\kern0.5em =\kern0.5em 1-{\Sigma}_i{\left({y}_i\kern0.5em -\kern0.5em {\widehat{y}}_i\right)}^2/{\Sigma}_i{\left({y}_i\kern0.5em -\kern0.5em {\overline{y}}_i\right)}^2. $$

Where *y*_*i*_ is the observed SF-6D index score for a subject, ${\overline{y}}_i$ is the mean of the observed SF-6D index scores, and ${\widehat{y}}_i$ is the predicted SF-6D index score for a subject. The predicted values are determined by applying the derived β coefficients from each MCMC simulation iteration to the observed SF-6D values.

In addition, we calculated the adjusted *R*^2^, which is a modification of the *R*^2^ that adjusts for the number of explanatory terms in the model. It is defined as

4 $$ \mathrm{Adjusted}\kern0.5em {R}^2\kern0.5em =\kern0.5em 1-\left\{\left(1-{R}^2\right)\ast \left[\left(n-1\right)/\left(n\kern0.5em -\kern0.5em p\kern0.5em -\kern0.5em 1\right)\right]\right\}. $$

where *n* is the sample size and *p* is the number of covariates in the model. An additional metric of model performance was the mean of the absolute prediction error, which is defined as the absolute difference between the predicted and observed value. As the intended purpose of our models is to predict the mean SF-6D scores, we also compared the predicted versus observed mean SF-6D scores in the overall data set.

The validity of candidate models according to the quality of their point estimates of utility can be estimated using root mean square error (RMSE) criterion for the mean:

5 $$ \mathrm{RMSE}\kern0.5em =\kern0.5em {\left({\Sigma}_{i=1}^T{\left({y}_i\kern0.5em -\kern0.5em {\widehat{y}}_i\right)}^2/n\right)}^{1/2} $$

#### Comparison of models

All presented models have their frequentist counterfactual and so the best performing Bayesian model will be compared to its frequentist counterfactual [11]. Given the overall aim is to predict health state valuation; the best way to compare these models is via their predictive ability. This includes plots of predicted to actual values, calculations of the mean predicted error, RMSE, R^2^/adjusted R^2^ and plots of the standardised residuals. These assessments are undertaken within the full estimation sample and in an out of sample random selection of 12 states by re-estimating the models using data sets excluding these 12 states.

## Results

The four Bayesian models are compared in terms of their coefficients with their appropriate 95%CI, and their predictive performance. Table 2 shows the intercept and β coefficients as well as the corresponding 95% credible intervals for each of the SF-6D components for all of the models. The posterior intercept for models 1 and 2 were 0.829 and 0.833 respectively, while the intercepts for models 3 and 4 were fixed to unity. For models 2, 3, and 4, representing the RE models, all coefficients have the expected negative sign. However, for model 1 (simple linear regression), the vast majority of coefficients have the expected (negative) sign. There are three positive coefficients associated with 3 levels and distributed over 2 dimensions, those being level 3 of the dimension *physical functioning* i.e. PF3, and levels 2 and 3 of the dimension *pain* i.e. PAIN2 and PAIN3. Further model 4 includes the dummy variable ‘most’ which take a value of 1 if any dimension in the health state is at the most level. The coefficient estimates suggest a further negative effect if any dimension is at the most severe level. Finally, a key important finding from Table 2 is associated with the smaller weight in absolute value obtained for PF5 in all models, for SF3 in three models (models 2, 3, and 4) and for VIT3 in three of the four models (models 1, 3, and 4). We consider this finding in more detail in the discussion section.

**Table 2:** Coefficients for OLS and RE models

	Model 1	Model 2	Model 3	Model 4	Model 4 (frequentist)
*β*	0.829 (0.771, 0.889)	0.833 (0.787, 0.878)	1	1	1
*β PF2*	-0.009 (-0.055, 0.037)	-0.022 (-0.052, 0.008)	-0.058 (-0.089, -0.027)	-0.050 (-0.081,-0.020)	-0.050 (-0.080,-0.020)
*β PF3*	0.008 (-0.037, 0.054)	-0.027 (-0.057, 0.004)	-0.050 (-0.081, -0.020)	-0.039 (-0.070,-0.007)	-0.038 (-0.069,-0.007)
*β PF4*	-0.034 (-0.082, 0.013)	-0.065 (-0.097, -0.033)	-0.087 (-0.121, -0.056)	-0.069 (-0.102,-0.036)	-0.069 (-0.101,-0.036)
*β PF5*	-0.031 (-0.077, 0.014)	-0.045 (-0.075, -0.014)	-0.060 (-0.092, -0.030)	-0.046 (-0.078,-0.014)	-0.046 (-0.077,-0.014)
*β PF6*	-0.114 (-0.161, -0.066)	-0.136 (-0.167, -0.104)	-0.160 (-0.193, -0.128)	-0.146 (-0.178,-0.114)	-0.145 (-0.177,-0.113)
*β RL2*	-0.023 (-0.060, 0.013)	-0.026 (-0.052, -0.001)	-0.055 (-0.079, -0.031)	-0.051 (-0.075,-0.027)	-0.051 (-0.074,-0.027)
*β RL3*	-0.035 (-0.074, 0.003)	-0.054 (-0.081, -0.027)	-0.075 (-0.100, -0.049)	-0.058 (-0.085,-0.031)	-0.058 (-0.085,-0.031)
*β RL4*	-0.034 (-0.074, 0.005)	-0.055 (-0.083, -0.028)	-0.077 (-0.103, -0.051)	-0.063 (-0.090,-0.036)	-0.063 (-0.090,-0.036)
*β SF2*	-0.015 (-0.055, 0.024)	-0.033 (-0.060, -0.006)	-0.065 (-0.091, -0.040)	-0.053 (-0.079,-0.027)	-0.054 (-0.080,-0.027)
*β SF3*	-0.041 (-0.082, 0.000)	-0.022 (-0.050, 0.005)	-0.047 (-0.074, -0.020)	-0.032 (-0.059,-0.004)	-0.032 (-0.059,-0.004)
*β SF4*	-0.047 (-0.087, -0.007)	-0.041 (-0.068, -0.014)	-0.065 (-0.091, -0.039)	-0.043 (-0.071,-0.015)	-0.044 (-0.071,-0.016)
*β SF5*	-0.084 (-0.127, -0.040)	-0.088 (-0.117, -0.059)	-0.109 (-0.138, -0.080)	-0.095 (-0.125,-0.066)	-0.096 (-0.125,-0.066)
*β PAIN2*	0.008 (-0.037, 0.054)	-0.001 (-0.031, 0.029)	-0.041 (-0.070, -0.013)	-0.037 (-0.066,-0.009)	-0.037 (-0.066,-0.009)
*β PAIN3*	0.004 (-0.038, 0.0487)	-0.017 (-0.046, 0.012)	-0.045 (-0.074, -0.016)	-0.034 (-0.063,-0.005)	-0.034 (-0.064,-0.005)
*β PAIN4*	-0.034 (-0.080, 0.010)	-0.025 (-0.056, 0.004)	-0.053 (-0.083, -0.022)	-0.040 (-0.071,-0.010)	-0.040 (-0.070,-0.009)
*β PAIN5*	-0.066 (-0.109, -0.021)	-0.067 (-0.097, -0.037)	-0.102 (-0.131, -0.073)	-0.081 (-0.110,-0.051)	-0.081 (-0.111,-0.050)
*β PAIN6*	-0.160 (-0.202, -0.118)	-0.154 (-0.184, -0.126)	-0.177 (-0.206, -0.149)	-0.167 (-0.196,-0.139)	-0.167 (-0.195,-0.138)
*β MH2*	-0.034 (-0.077, 0.008)	-0.018 (-0.047, 0.011)	-0.043 (-0.072, -0.015)	-0.036 (-0.064,-0.007)	-0.036 (-0.065,-0.007)
*β MH3*	-0.026 (-0.069, 0.016)	-0.032 (-0.062, -0.002)	-0.055 (-0.085, -0.025)	-0.046 (-0.075,-0.015)	-0.045 (-0.076,-0.015)
*β MH4*	-0.098 (-0.142, -0.054)	-0.093 (-0.122, -0.062)	-0.116 (-0.145, -0.086)	-0.100 (-0.130,-0.070)	-0.099 (-0.130,-0.069)
*β MH5*	-0.131 (-0.176, -0.087)	-0.106 (-0.136, -0.075)	-0.125 (-0.156, -0.095)	-0.116 (-0.146,-0.085)	-0.115 (-0.147,-0.084)
*β VIT2*	-0.044 (-0.086, -0.009)	-0.005 (-0.034, 0.023)	-0.038 (-0.065, -0.011)	-0.031 (-0.058,-0.003)	-0.032 (-0.058,-0.004)
*β VIT3*	-0.035 (-0.080, 0.007)	-0.007 (-0.037, 0.022)	-0.029 (-0.058, 0.000)	-0.018 (-0.048,0.012)	-0.019 (-0.048,0.012)
*β VIT4*	-0.033 (-0.077, 0.010)	-0.010 (-0.040, 0.020)	-0.038 (-0.066, -0.010)	-0.021 (-0.051,0.009)	-0.022 (-0.051,0.008)
*β VIT5*	-0.077 (-0.122, -0.0034)	-0.067 (-0.098, -0.036)	-0.086 (-0.115, -0.057)	-0.072 (-0.103,-0.042)	-0.073 (-0.103,-0.043)
*β most*	NA	NA	NA	-0.083 (-0.118,-0.048)	-0.084 (-0.119,-0.048)
*σ*	0.347 (0.339, 0.355)	0.218 (0.212, 0.223)	0.219 (0.214, 0.225)	0.219 (0.214,0.225)	NA

*PF* physical functioning, *RL* role limitations, *SF* social functioning, *PAIN* pain, *MH* mental health, *VIT* vitality, *NA* not applicable. Values given as posterior mean (95% confidence interval). The number next to each parameter (2, 3, 4, 5, and 6) refers to the level within each dimension

The levels of performance of the models are summarized in Table 3, by showing their appropriate R^2^, adjusted R^2^, mean predicted error, and RMSE. Models 2, 3 and 4 proved to be very similar by having values variating at the level of the third decimal, where they are all responsible to explain about 68% of the variation in the data, based on their R^2^ and adjusted R^2^, while model 1 could explain around 20% of the variation in the data. Additionally, the mean predicted error for model 1 is computed to be around 0.28 while those of the other 3 models are smaller with a value of 0.17. Further, based on the RMSE, models 2, 3 and 4 performed much better than model 1, with values of 0.22 and 0.35 respectively, hence the error in models 2, 3 and 4 is smaller than in model 1. Overall, model 4 was found to provide the best fit to the data (with mean predicted error of 0.166, R^2^ of 0.683, adjusted R^2^ of 0.681 and RMSE of 0.218) when compared to models 2 and 3. On that basis we proceed the analysis with model 4 as the best performing Bayesian model in this paper. To this end, model 4 was also found to provide a slightly better fit to the data when compared to its frequentist counterfactual (with mean predicted error of 0.167, R^2^ of 0.682, adjusted R^2^ of 0.680 and RMSE of 0.219).

**Table 3:** Performance of the Bayesian models

	R^2^	Adjusted R^2^	Mean predicted error	RMSE
Model 1	0.197	0.191	0.283	0.347
Model 2	0.679	0.677	0.168	0.219
Model 3	0.681	0.679	0.167	0.218
Model 4	0.683	0.681	0.166	0.218
Model 4 (Frequentist)	0.682	0.680	0.167	0.219

*R*^*2*^ proportion of the variance as explained by the model, *RMSE* Root Mean Square Error

**Fig. 1:**
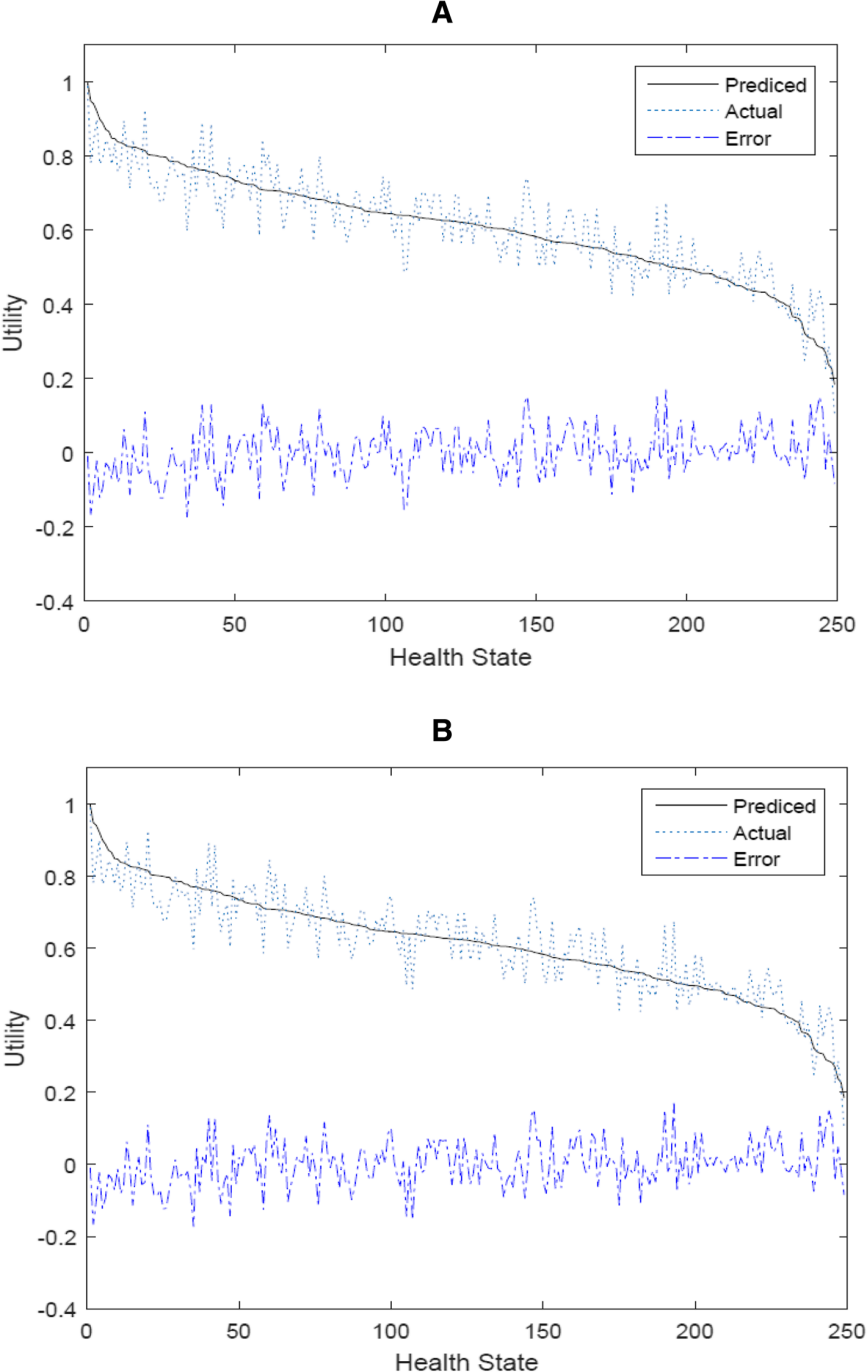
Actual and predicted mean health states valuations for (**a**) the Bayesian model (model 4) and (**b**) the frequentist model

The model has been tested in terms of its predictive ability, where the predicted and actual mean values for the 249 health states valued by the representative population have been plotted with health states ordered by predicted health state values.^3^ Figure 1a represents the resulting predicted mean health state valuation, solid line, alongside the actual mean health state valuations, represented by the dotted line. Additionally, the dashed line represents the errors obtained by the difference between the two valuations. It is clear that the model predicts the data quite well for all heath states. For comparison, Fig. 1b presents the corresponding plots for the frequentist model (final column of Table 2). Although the results of the frequentist model 4 are comparable to the results of Bayesian model 4 in Fig. 1, the Bayesian model has the advantage of providing full probability distributions of the 249 health state utilities as a direct output from the modeling process rather than simply providing the mean value and/or standard deviation as is the case with the frequentist model. It may be argued that the frequentist model can do this, but only after producing the distribution of the betas and then using a Cholesky approach to derive health states distributions. These distributions are hugely important to capture the full range of uncertainty inherent in these utility estimates -- an increasingly important input to cost effectiveness analyses for health technology assessment. More about this point will be discussed later in the article.

**Fig. 2:**
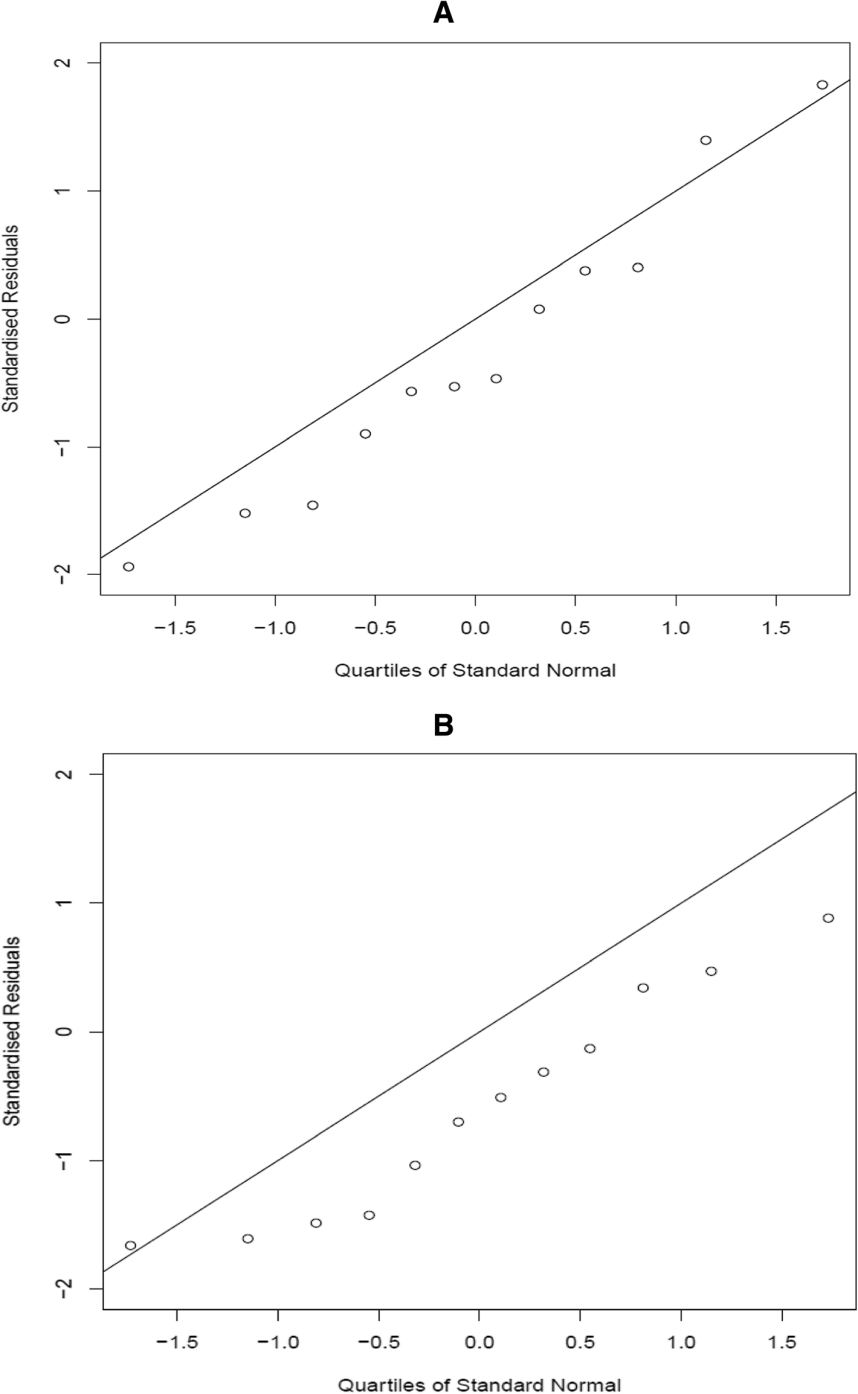
Q-Q plot of standardised predictive errors for the 12 out of sample health states for (**a**) the Bayesian model (model 4) and (**b**) the frequentist model

As always, it is important to test the validity of the Bayesian model through investigating its ability in predicting the values for states that haven’t been used in the initial estimation. Therefore, we excluded data related to 12 health state values randomly from the initial 249 health states data set, and the Bayesian model was fitted on the remaining 237 SF-6D health states. The results are summarized in Table 4, showing the true sample means for the 12 omitted health states, along with their predicted posterior means and SDs from the Bayesian model, and comparing them to their actual mean utility values. It can be seen that the predicted health state utility values for the 12 omitted health states generated by the Bayesian approach turned out to be very close to the actual mean utility for the appropriate health state with very small differences. For instance, the predicted mean utility value for health state 112,111 was predicted to be 0.8704 whereas the actual mean value is 0.8212, similarly for health state 325,455, the predicted and actual mean values were respectively 0.4612 and 0.4677. Whilst for other states, we see that the difference between estimates is large (e.g. for state 621,221). To better assess the predictive performance of the Bayesian model, Fig. 2a shows a Q-Q plot of the standardized prediction errors for the 12 omitted health states. The straight line in the figure corresponds to the theoretical *N*(0,1) distribution. Based on this model, we expect to observe the quantiles of the standardized prediction errors to lie roughly on the theoretical line. As can be seen, the values are at enough proximity from the line to validate the model’s predictions.

**Table 4:** Out of sample predictions for 12 health states

Health state	True sample mean	Bayesian Posterior Inference			Frequentist Inference		
		Mean	S.D.	Difference	Mean	S.D.	Difference
112111	0.8212	0.8704	0.0867	-0.0492	0.9442	0.1184	-0.123
133132	0.5686	0.7400	0.0884	-0.1714	0.7508	0.1096	-0.1822
223451	0.6755	0.5122	0.0892	0.1633	0.6107	0.1366	0.0648
235224	0.4687	0.6050	0.0896	-0.1363	0.6331	0.1105	-0.1644
241531	0.7534	0.6282	0.0894	0.1252	0.6743	0.0897	0.0791
325455	0.4677	0.4612	0.0883	0.0065	0.4985	0.0977	-0.0308
332411	0.7692	0.7353	0.0896	0.0339	0.7329	0.1061	0.0363
333154	0.6364	0.6013	0.0879	0.0351	0.6482	0.0890	-0.0118
421314	0.7121	0.7597	0.0898	-0.0476	0.7658	0.1057	-0.0537
423433	0.5759	0.6172	0.0878	-0.0413	0.6564	0.1141	-0.0805
545644	0.2484	0.3269	0.0872	-0.0785	0.4295	0.1268	-0.1811
621221	0.4937	0.6235	0.0889	-0.1298	0.6581	0.1020	-0.1644

**Fig. 3:**
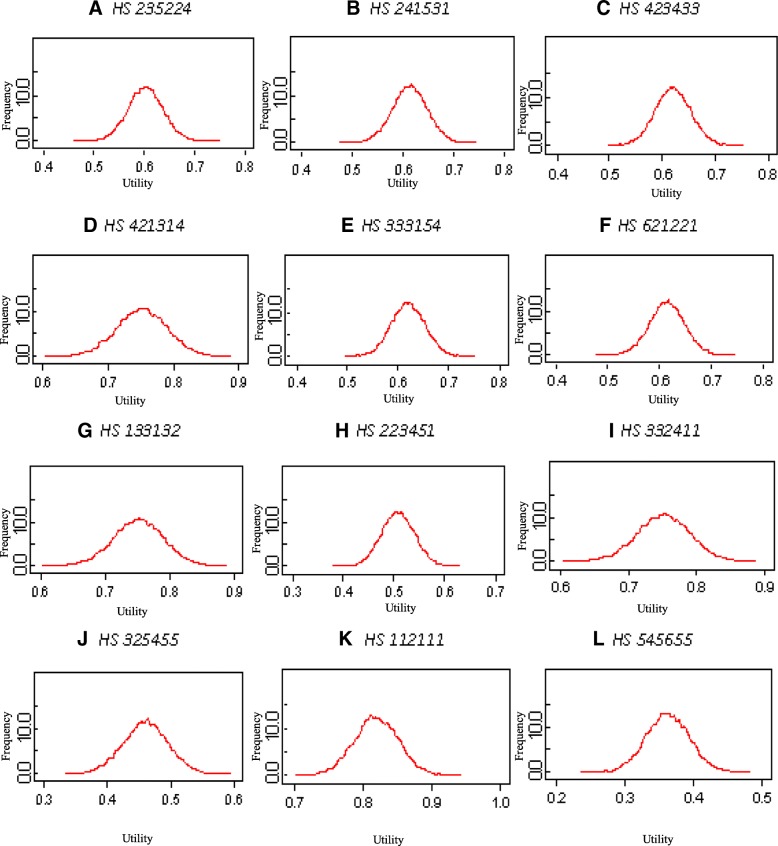
Probability Distribution around the predicted utility value for each of the 12 omitted health states

The final two columns of Table 4 presents then means and SDs predictions that were obtained from the reduced data but fitting the frequentist model. The predictions are generally less accurate than those which were produced from the Bayesian model. In particular, across the 12 omitted health states, the RMSE of predication is 0.100 for the Bayesian model estimates and 0.114 for the frequentist model. In addition, Fig. 3b shows the corresponding Q–Q plot for the frequentist model. In Fig. 2b, the points deviate substantially and systematically from the theoretical line; therefore, the frequentist model is not well validated by its predictive performance. In contrast, it is apparent from Fig. 2a that the Bayesian model predictions are well validated. Note that the difference in predictions is primarily because the Bayesian analysis is able to make use of other evaluations by the same respondents to estimate their individual random effects, which the frequentist analysis cannot do.

Finally, a key advantage of the Bayesian method is that it provides estimates of the uncertainty in the health state predictions from the model. On the other end, the frequentist model provides data on the uncertainty in the model parameters, but they do not provide estimates of the uncertainty in the health state predictions from the model [23, 24]. Figure 3 shows the probability distribution around the predicted utility value for each of the 12 omitted health states. From these distributions, the mean, median, standard deviation and corresponding 95% credible intervals can be calculated. This leads to a conclusion that the Bayesian method is more flexible in characterizing inputs to regression models and more comprehensive in characterizing the uncertainty in the model outputs [23, 24].

## Discussion

The aim of this paper is to put on the map of modelling a new approach to estimating SF-6D health state utility values using Bayesian methods. This will help health care professionals in deriving better health state utilities of the original UK SF-6D for their specialized applications. After analyzing several models using the Bayesian approach, we found that by adopting the model including the random effect of the individual, in addition to the inclusion of the additional parameter ‘most’ and fixing the intercept to unity, we would be able to predict health state values that are very close to the actual values given by the respondents in their valuations with near absent over/under estimation. This best fitted model is responsible for explaining about 68% of the variation in the data with very low error, RMSE of 0.21.

An issue of note regarding the existence of inconsistencies between coefficients on the SF-6D levels. Those inconsistencies that occur in more than one of the four models reported in Table 2 are as follows: PF4 versus PF5, SF2 versus SF3 and VIT2 versus VIT3. PF4 versus PF5 have similar coefficients across all models and this indicates that most respondents did not distinguish between them. For SF2 versus SF3 and VIT2 versus VIT3 one possible explanation is that this dimension is worded in the positive rather than the negative and this may have caused some confusion for respondents. We do not believe these inconsistencies have any serious implication for the performance of the model as whole except for a reduction in sensitivity at the upper end for some dimensions. Of course, a larger sample size and the valuation of additional health states may have overcome some of these problems [11]. Analysis by Brazier et al. [11] has also found this type of inconsistencies.

Through employing Bayesian methods to proceed with our works, we were able to incorporate parameter estimation uncertainty in our results. Conventional approaches to the assessment of health state utility models; both direct and indirect utility models, have considered three broad factors for model assessment and selection: how good the models are in fitting the data; the conventional psychometric properties of the utility algorithms based upon the models, and the accuracy of the predictive performance of the models [23, 24]. These factors are suitable only in the case when decision makers are interested in the models’ expected value predictions. However, when they are interested in decision uncertainty and include research within their decision options, the precision of the model predictions becomes a fourth factor related to the assessment and selection of health state utility models [23, 24]. In this context, we would argue that Bayesian models such as those presented in the paper are superior to the frequentist equivalents as they are able to produce information on the predictive performance precision as a direct output from the modeling process. Hence, Model 4 performs best in this paper as it makes maximum use of the available information and predicts more accurately. More importantly, it provides the information necessary to establish the value of undertaking further research on the utility parameters in any decision analysis it informs.

In this article we have focused on the use of linear random effect models for predicting health states utilities. However, other strategies showing great predictive abilities compared to the model adopted in this study may be employed. Those include generalized linear models, Tobit models, Two-part models and survival-type models. While the linear regression model is the most widely adopted type of predictive models, showing great power in the application despite their theoretical limitations, the literature evidently shows that the ideal model depends extensively on the data on hand.

## Conclusion

In conclusion, this paper has proposed four alternative random effects models for modelling and predicting utilities. The analyses presented have demonstrated how utility data may be straightforwardly modelled using Bayesian methods, and model fit and complexity assessed using R^2^/adjusted R^2^ and RMSE, which are straightforward to compute in a MCMC analysis. The Bayesian models are able to produce probability distributions as a direct output from the modeling process describing the uncertainty in the expected health state values - an increasingly important input to cost effectiveness analyses for health technology assessment. We hope that this work will provide applied researchers with a practical set of tools to appropriately model outcomes in CEA.

## Abbreviations

AQoL: Assessment of Quality of Life
CEA: Cost-effectiveness analyses
EQ-5D: The EuroQol
HRQoL: Health related quality of life
HUI: Health Utilities Index
MAU: Multi-attribute utility
MCMC: Markov chain Monte Carlo
MH: Mental Health
N(.,.): Normal distribution
OLS: Ordinary least squares
PAIN: Pain
PF: Physical Functioning
QALYs: Quality adjusted life years
Q-Q plot: Quantile-Quantile plot
R^2^: Proportion of the variance as explained by the model
RE: Random effect
RL: Role Limitations
RMSE: Root mean square error
SD: Standard deviation
SF: Social Functioning
SF-36: Short form health survey
SF-6D: Short form 6 dimensions health survey
SG: Standard gamble
UK: United Kingdom
VIT: Vitality
WinBUGS: Statistical software for Bayesian analysis using MCMC

## Footnotes

1 As mentioned in Brazier et al. [11], these were addresses which contained no resident household for various reasons including: insufficient address, not traced, not yet built, derelict/demolished, business only, empty, institution only, weekend/holiday home.

2 The remaining 442 were non-responders. Their addresses were correct and their doors knocked on but they either were not home or said they did not want to participate.

3 The graphs in Figure 1 have been plotted by ordering states in terms of their predicted values rather than observed values as presented in Brazier et al. [11]. This is in line with regression analysis where the actual observations are represented by circular dots and the best fit or predicted regression line is represented by the diagonal solid line.
